# Adsorption Behavior and Mechanisms of Trihalomethanes onto Virgin and Weathered Polyvinyl Chloride Microplastics

**DOI:** 10.3390/toxics12070450

**Published:** 2024-06-22

**Authors:** Yi Li, Paragi Neema, Susan Andrews

**Affiliations:** Department of Civil & Mineral Engineering, University of Toronto, Toronto, ON M5S 1A4, Canada; paragi.neema@mail.utoronto.ca (P.N.); susan.andrews@utoronto.ca (S.A.)

**Keywords:** microplastics, PVC, THMs, disinfection byproducts, interactions

## Abstract

Microplastics that adsorb various toxic contaminants in water may be transported into cells and organs, possibly posing toxicological risks in the aquatic environment. Disinfection byproducts (DBPs), which are ubiquitous in chlorinated drinking water and wastewater, may have some potential to sorb onto microplastics (MPs) through hydrophobic or electrostatic interactions. However, DBP adsorption on microplastics has not yet been closely examined. This work investigated the adsorption behavior of trihalomethanes (THMs)—a regulated and ubiquitous DBP class in chlorinated water—onto virgin and weathered polyvinyl chloride (PVC) microplastics, the most widely used plastic material in drinking water distribution and sewer systems. A comparative analysis of kinetic and isotherm test results indicated that the adsorption mechanisms mainly involved hydrophobic interactions from a combination of weak and strong physisorption behavior and possibly chemisorption. The adsorption coefficients from all the models examined suggested that the adsorption of THMs, and perhaps chemically similar DBPs, onto virgin PVC microplastics can be 10–20 µg g^−1^. However, the weathered PVC microplastics contained more polar functional groups, which led to a decreased hydrophobicity and reduced THM adsorption capacity by approximately 10%. These findings offer novel insights into the possible adsorption characteristics of disinfection byproducts (DBPs) onto microplastics and will assist in targeting more toxic DBPs for future investigations.

## 1. Introduction

Microplastics (plastic debris <5 mm in size), as a type of emerging contaminant, are globally present almost in all aquatic media, ranging from the ocean to freshwater lakes and rivers [1,2,3,4,5,6], and are being increasingly reported in municipal and bottled drinking water, as well as treated wastewater [7,8,9,10]. The health risks linked to microplastics (MPs) in water are a growing concern, since they have been reported in human blood and in the tissues of aquatic animals such as fish and mussels [11,12,13]. Due to their high specific area and intrinsic hydrophobicity, MPs also act as potential carriers of organic micropollutants. Although MPs can be removed by up to 99% in drinking water and wastewater treatment plants [8,9,10,14], from hundreds to thousands of particles per liter of microplastics can remain in the treated water. Thus, the intake of microplastics and potential uptake of associated contaminants into the human body and aquatic animals have received increasing attention.

Frequently observed polymer types include polyethylene (PE), polypropylene (PP), polystyrene (PS), polyethylene terephthalate (PET), and polyvinyl chloride (PVC), which roughly agrees with the order of their production volumes [15]. Although most types of microplastics in surface water may differ by location, and be influenced to differing degrees by upstream inputs of treated wastewater, PET, PE, and PP are reported as the dominant types in drinking water treatment plants (DWTPs) in Czechia [8] and China [10]. Other types of microplastics, such as PVC and PS, were also found in all water samples by Wang et al. (2020) [10]. Despite its relatively low occurrence in treated water, PVC is of particular concern as the predominant plastic material in drinking water distribution and sewer systems.

Currently, the extent of contaminant adsorption onto microplastics in treated water remains unclear. Previous research on organic/inorganic compound adsorption onto microplastics has primarily focused on the pollutants found in diverse water sources, with limited focus on the compounds present in treated water, notably, disinfection byproducts (DBPs). For example, in various aquatic environments, microplastics have been shown to sorb organic pollutants of different classes, such as antibiotics, pesticides, polycyclic aromatic hydrocarbons (PAHs), and dissolved organic matter (DOM) [16,17,18,19,20].

In treated water, the adsorption of disinfection byproducts (DBPs) onto microplastics should not be ignored, since microplastics that escape treatment may still have the potential to adsorb various DBPs. As MPs may concentrate contaminants in the particles, tiny microplastic particles (e.g., nanoplastics) that escape removal may cross cell membranes and enter various tissues to deliver higher concentrations of toxic DBPs to cells and organs than they would typically be exposed to. The toxic DBPs at higher concentrations in these cells and tissues may have potential toxicological risks to human and aquatic life.

Although trihalomethanes (THMs) are not the most toxic in the complex mixture of DBPs, some epidemiologic studies have shown that THM levels are associated with bladder and rectal cancers [21,22]. Conventional water treatment methods have been shown to be inadequate for removing THMs, which remain in the distribution systems and pose potential health risks to consumers [23,24,25,26]. The physical–chemical properties of THMs suggest that they may have some potential to sorb onto microplastics through hydrophobic or electrostatic interactions. Therefore, in addition to the risks caused by THMs or microplastics separately, there also may be health risks associated with THMs delivered to cells and tissues by tiny microplastics. Regarding the interaction between microplastics and DBPs, some studies have indicated that microplastics can release dissolved organic matter (DOM) as DBP precursors [27,28]. Yan et al. (2024) demonstrated that microplastics can act as sources and sinks of both DOM and DBPs [29]. However, these studies either concentrate on the role of microplastics as sources or on a single species of THMs (i.e., chloroform) or other polymer types such as PP or PS. Thus, the interactions between THMs and PVC MPs in water have not yet been extensively investigated.

Weathered microplastics in the natural environment may exhibit different adsorption behavior compared to virgin particles due to physical and chemical changes from wear and tear, sunlight, and temperature variations [30,31,32]. Weathered microplastics often develop rougher surfaces, larger surface areas, and more oxygen-based groups on their surfaces, making their surfaces more polar or hydrophilic than those of corresponding virgin polymers [30,31,32]. Although previous research has indicated that weathered microplastics exhibit enhanced adsorption for pharmaceuticals and natural organic matter [30,33,34,35], limited research has addressed the impact of weathered polymers, particularly PVC, on organic contaminant adsorption in chlorinated water.

In this study, bench-scale kinetic and isotherm adsorption tests were conducted to investigate the adsorption of THMs (chloroform, bromodichloromethane, chlorodibromomethane, and bromoform) onto virgin and weathered microplastics. The linear least-squares method was applied to assess the suitability of different sorption models for the kinetic and isotherm data. Preliminary experimental results (Table S1 and Figure S1) demonstrated that polyethylene (PE), polypropylene (PP), polyamide (PA), polyacrylonitrile (PAN), and polyethylene terephthalate (PET) microplastics have a low adsorption capacity for THMs (<0.1 µg g^−1^ for PE, and <5 µg g^−1^ for the other microplastics). In comparison, PVC has shown greater adsorption (>10 µg g^−1^) of THMs, which may be related to the affinity of the similar structural elements, i.e., C-Cl bonds, that exist in both THM and PVC molecules. In addition, the more porous surface of PVC microplastics, as shown in surface characterization tests, may also contribute to their higher adsorption of THMs than other polymers (Figure S2).

The objective of this research was twofold: (1) to explore the possible sorption mechanisms of THMs onto PVC microplastics by applying various kinetic and isotherm models to the experimental data; and (2) to assess the impact of weathering on microplastic adsorption behavior by comparing the sorption capacities for virgin and weathered PVC microplastics. Although THMs are often regarded as less toxic than other DBPs in drinking water, they are frequently used as surrogates for the occurrence or removal of other DBPs that are more toxic and difficult to measure. This research sought to gain valuable insights into these adsorption characteristics and serve as an aid for future investigations concerning more toxic DBPs.

## 2. Materials and Methods

### 2.1. Materials

White PVC microplastic powder (molecular weight ~90,000, oval to spherical shape, as shown in Figure S2a) was purchased from Sigma-Aldrich (Oakville, ON, Canada). The particle size range used in the experiments was 125–300 µm after sieving by stainless-steel sieves with mesh sizes of 45, 125, and 300 µm (Cole-Parmer, Montreal, QC, Canada). Although studies have indicated that microplastics larger than 150 μm are likely to be expelled directly through feces from the human body [36], the contaminants accumulated on them may have adverse impacts on human health while they are within the body.

Weathered microplastics were prepared in continuously aerated and irradiated batch reactors for eight weeks. This method was adapted from Andrade et al. (2019) [37], which accounts for both the hydrolytic and photo oxidative weathering of MPs under realistic exposure conditions [32,38]. In brief, each 1 L borosilicate glass cylinder held from 10 to 20 g of microplastics (MPs), along with 750 mL of Elix^®^ water ((Millipore Sigma, Burlington, MA, USA) and 100 mL of silica sand. Sunlight simulation was achieved using a 600W MH metal halide lamp (Daylight Blue, Hortilux, Mentor, OH, USA), while mechanical abrasion was facilitated by aeration and standard filter sand (Anthrafilter Media & Coal Ltd., Brantford, ON, Canada).

Reference material-grade Trihalomethanes (THMs) Calibration Mix (2000 mg L^−1^ in methanol) was purchased from Sigma-Aldrich (Oakville, Canada). The mix solution contained four regulated THM compounds, including chloroform (trichloromethane, TCM), bromodichloromethane (BDCM), dibromochloromethane (CDBM), and bromoform (tribromomethane, TBM). The purchased THM Mix solution was then diluted to 200 mg L^−1^ in methanol as the intermediate stock solution and injected into the adsorption test bottles to reach a target concentration of 100 µg L^−1^ for each compound. Although the current maximum acceptable concentration (MAC) is 100 µg L^−1^ for total THMs in Canada, concentrations of individual THM species have been observed near the MAC in some chlorinated waters [39]. In addition, the initial concentrations were 100 μg L^−1^ so that the remaining concentrations after adsorption would be above the method detection limits (MDLs, generally ~0.5 µg L^−1^) and still allow for changes in concentration of a few orders of magnitude during the adsorption test. Only 125 µL of the solution in methanol was added to each 250 mL bottle (<0.2%) to achieve the target concentration without a co-solvent effect. Reagent-grade sodium azide (NaN_3_) was purchased from Sigma-Aldrich (Oakville, ON, Canada).

High-performance liquid chromatography (HPLC)-grade methanol was purchased from Fisher Scientific (Whitby, ON, Canada). For THM extraction, HPLC-grade Methyl tert-butyl ether (MTBE) was purchased from JT Baker (Med Store, Toronto, ON, Canada), and ACS reagent-grade sodium sulphate (Na_2_SO_4_) was from Fisher (Whitby, ON, Canada). This study used ultrapure water (18.2 MΩ cm^−1^) treated by a Milli-Q^®^ (MQ) water purification system (Millipore Sigma, Burlington, MA, USA). The glassware was baked at 250 °C for at least 6 h before use.

### 2.2. Batch Adsorption Experiments

Adsorption kinetics tests were conducted using 1 g of PVC microplastics, 100 µg L^−1^ of THMs, and 25 mg L^−1^ of NaN_3_ as a bioinhibitor in amber glass bottles filled with 250 mL of MQ water headspace free. The dose of PVC was determined from preliminary tests so that both THM reduction was measurable and the THMs remained in measurable amounts at the end of the tests. An initial test was conducted to confirm that 25 mg L^−1^ of NaN_3_ can maintain the THM concentrations but does not interfere with THM–PVC microplastic interactions (Figure S3 and Table S2). The bottles were put into rotating mixers at 22 ± 1 °C for 18 intervals up to 35 days. Samples were collected at 6, 12, 24, 36, 48, 60, 72, and 96 h in the first four days and twice every week until 35 days. The samples collected at each time interval were passed through a 45 µm pore size stainless-steel sieve and a glass funnel to stop the adsorption process, and only the liquid phase was collected to measure the remaining THM concentrations. The filtered samples were collected in 40 mL amber glass vials headspace free and stored at 4 ± 2 °C until extraction with MTBE and a GC-ECD analysis for THMs (Section 2.3). Each sample was both prepared and extracted in duplicate for analysis.

The amount of THMs adsorbed onto a per unit mass of microplastics at time t (day), q_t_ (µg g^−1^), was calculated by the mass balance equation as follows:

(1) $$q_{t}=\frac{\left(C_{0}-C_{t}\right)V}{m}$$

where C_0_ and C_t_ are the THM concentrations at time 0 and t in the liquid phase, respectively (µg L^−1^); m is the mass of solid phases (g); and V is the volume of the solution for the adsorption test (L).

Adsorption isotherm experiments were conducted with PVC doses ranging from 50 mg to 2.5 g in 250 mL of water. Although varying the initial contaminant concentrations has been the more traditional method used in adsorption studies, changing the dose of the adsorbent has also been adopted in many research papers [40,41,42]. The PVC microplastics were added into 250 mL amber glass bottles filled headspace free with MQ water containing 100 µg L^−1^ of THMs and 25 mg L^−1^ of NaN_3_. Based on the kinetics experiments, the duration was 21 days for the adsorption test to reach equilibrium. Samples were collected and stored following the same procedures for the kinetics test.

### 2.3. Analytical Methods

The four THMs were analyzed according to the laboratory-developed method based on Standard Method 6232 [43] and EPA551.1 [44]. The concentrations of THMs were determined by the liquid–liquid extraction with MTBE and gas chromatographic method. The gas chromatograph used for this analysis was an Agilent 7890B Series Gas Chromatograph using an electron capture detector (GC-µECD) and a DB 5.625 capillary column (1225631, Agilent Technologies Canada Inc., Mississauga, ON, Canada).

The surface morphology of the MP particles was observed with a Hitachi SU 7000 Ultra-High-Resolution Schottky SEM scanning electron microscope (SEM), and the functional groups present were identified by attenuated total reflectance-Fourier transform infrared spectroscopy (ATR-FTIR, Perkin Elmer, Waltham, MA, USA). The FTIR spectra were obtained over a range of 4000–400 cm^−1^ with a resolution of 0.482 cm^−1^, 30 scans per sample. The samples were analyzed in duplicate. All spectra were background-corrected, ATR-corrected, and baseline-corrected. Elemental analysis was performed by a K-alpha X-ray photoelectron spectroscopy (XPS) system (ThermoFisher Scientific, East Grinstead, UK) equipped with a monochromatized Al K-Alpha X-ray source and a 300 μm spot size (2:1 ellipse).

### 2.4. Adsorption Kinetics and Isotherm Models

The experimental kinetic data were fitted to pseudo-first-order and pseudo-second-order kinetic models. The pseudo-first-order model assumed that the time-based adsorption rate was proportional to the difference between the amount adsorbed at each time (q_t_) and the adsorption capacity at equilibrium (q_e_) [45]. The pseudo-second-order model assumed that the adsorption rate was controlled by the chemical bond formation between the adsorbent and the adsorbate, which involves sharing or exchanging electrons, creating valence forces in the chemisorption process [46,47]. Intra-particle diffusion, the Boyd kinetic model, and a diffusion–chemisorption kinetic model were also applied to investigate the adsorption mechanisms of THMs onto PVC (Table S3).

The isotherm experimental data were fitted to linear, Freundlich, Langmuir, and modified Freundlich models (Table S4). The modified Freundlich model accounts for a variable adsorbent dose [48,49]. The Results and Discussion section provides a more detailed explanation of these models.

### 2.5. Quality Control and Statistical Analysis

The loss of THMs in the experiment was evaluated by including microplastic-free controls with the same THM concentrations as the test samples. THM-free controls with microplastics only in MQ water were also included to confirm that the virgin PVC microplastics did not contain any THMs. Blanks with MQ water only were prepared to ensure that no THM contamination occurred in the experiment. The controls and blank samples were rotated for the same period as the test samples during the experiments, collected, and analyzed following the same procedures.

Experiments were run in duplicate. The linear least-squares method was used to determine the adsorption kinetic and isotherm model parameters in Microsoft Excel 2016 (Microsoft, Redmond, WA, USA).

## 3. Results and Discussion

### 3.1. Surface Characterization of Virgin and Weathered PVC MPs

**Figure 1:**
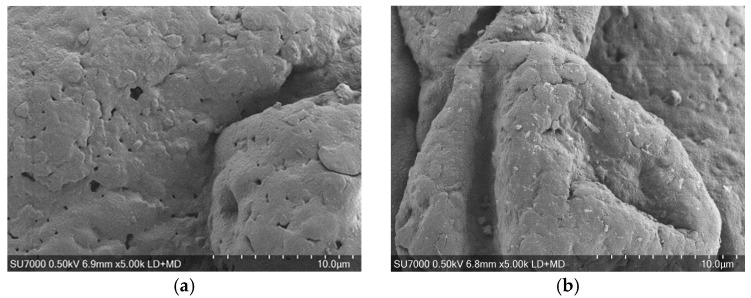
SEM images of virgin (**a**) and weathered (**b**) PVC microplastics.

According to the SEM analysis (Figure 1), the weathered PVC MPs showed more microcracks, pits, and grooves on their surfaces than the virgin particles, thus increasing the surface area of the microplastics. The elemental analysis by XPS demonstrated that the weathered PVC particles’ O/C ratio increased from 21% to 32%, whereas their Cl/C ratio decreased from 24% to 23% (Table S5). The FTIR results (Figure S4) revealed that the weathered PVC samples exhibited enhanced absorption bands in the regions of 1750–1690 cm^−1^ and 1140–940 cm^−1^, indicating an increased abundance of carbonyl groups (C=O) and carbon–oxygen bonds (C-O), respectively [50]. The increase in oxygen-containing functional groups like hydroxyl and carbonyl with weathering reduced the MPs’ hydrophobicity [30,35,51]. Although not measured in this study, weathering may also deteriorate the mechanical and thermal properties of microplastics through polymer cleavage, accompanied by changes in crystallinity characterized by reduced amorphous entanglements and polymer rearrangement, as reported in previous studies [30,31,35].

### 3.2. Adsorption Kinetics and Their Possible Relationships to Reported Mechanisms

Previous studies have shown that TCM adsorption onto polypropylene (PP) and polystyrene (PS) microplastics reaches equilibrium within 48 h at an initial concentration of 190 µg L^−1^ and a microplastic dose of 1 g L^−1^ [29]. Similarly, the adsorption of THMs onto crosslinked PE pipes also approached equilibrium within 48 h [52]. To the best of the authors’ knowledge, the THM adsorption equilibrium onto PVC microplastics has not been previously reported. In this study, the kinetic test results suggested that the adsorption tend to approach equilibrium by 21 days. The longer equilibrium times observed in this study may have been due to differences in the polymer types and particle sizes. Changes in THM concentrations during the kinetic test are presented in Figure S5.

**Figure 2:**
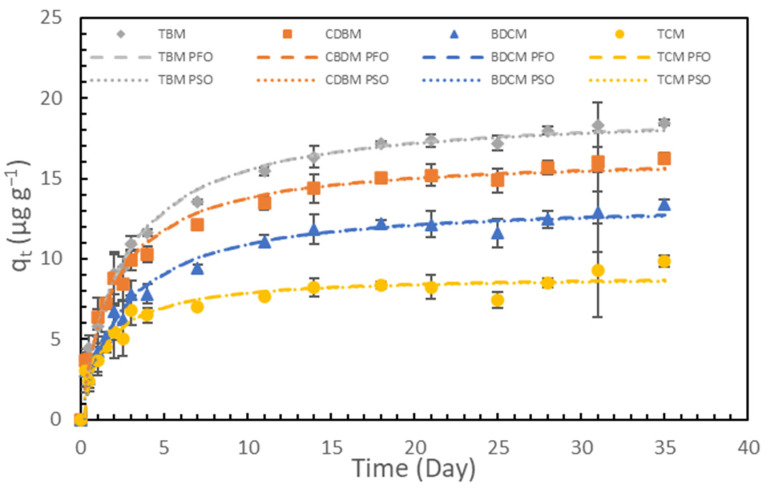
Experimental data and fitted curves for adsorption of THMs onto PVC with pseudo-first-order (PFO) and pseudo-second-order (PSO) kinetic models. Error bars represent experimental errors for duplicate analyses of duplicate samples.

The experimental data from the adsorption kinetics tests were first fitted with nonlinear forms of the standard pseudo-first-order and pseudo-second-order kinetic models to obtain estimates of q_e_, the time to reach equilibrium, and some indications of fundamental adsorption mechanisms (Figure 2). The pseudo-first-order and pseudo-second-order models both fit the data well (R^2^ > 0.9, except for the pseudo-first-order model for TCM, with R^2^ = 0.845). High R^2^ values for the pseudo-second-order models suggested that chemisorption may influence THM adsorption; however, they did not conclusively establish its occurrence [46]. While chemisorption is proposed as a likely mechanism, confirming its occurrence necessitates additional studies such as thermodynamic assessments, spectroscopic analyses for chemical interaction detection, and evaluations of changes in surface properties after adsorption. Regardless of the slight difference in R^2^, the predicted q_e_ values for the THMs and model fits for the two kinetic models were in excellent agreement, indicating that both physisorption and chemisorption could be occurring simultaneously or in different stages of the adsorption process. According to both models, the amount of adsorption increased by less than 1% after 21 days, suggesting that the adsorption process reached equilibrium within 21 days. The adsorption rate constants for the non-linear pseudo-first-order and pseudo-second-order models are summarized in Table 1.

The adsorption rates and q_e_ increased with the increasing hydrophobicity of the THM compounds (see, for example, the solubility data shown in Table S6), highlighting the substantial impact of hydrophobic interactions on THM adsorption onto PVC. A recent study reported similar observations for the adsorption of haloforms onto granular activated carbon (GAC) [25]. Other previous studies have also suggested that hydrophobic interactions are one of the primary mechanisms for the adsorption behavior of hydrophobic contaminants onto microplastics [53,54,55,56].

**Figure 3:**
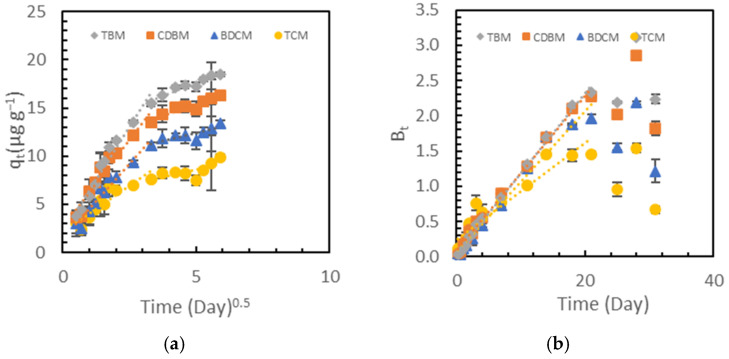
Experimental data and fitted curves for the adsorption of THMs onto PVC with (**a**) intraparticle diffusion model and (**b**) Boyd kinetic model. Error bars represent experimental errors for duplicate analyses of duplicate samples.

To further investigate the possible adsorption mechanisms and rate-limiting step(s) for the adsorption of THMs onto PVC, three kinetic models were used: intraparticle diffusion (Figure 3a), Boyd (Figure 3b), and diffusion–chemisorption (Figure S6) models. The adsorption coefficients are presented in Table S7.

The intraparticle diffusion model [57] assumes that adsorption occurs through a series of steps, starting from the mass transfer of the adsorbate from the bulk solution to the external surface of the adsorbent, followed by the diffusion of the adsorbate into the pores of the adsorbent [58]. This model can help to identify the rate-limiting step and the factors affecting the adsorption process. The model is characterized by parameters (Table S7) that include the intraparticle diffusion coefficient (k_p_) and the intercept (C), which are indicative of the rate of diffusion and the boundary layer effect, respectively. The larger intercept (C) indicates the greater contribution of surface mass transfer during the adsorption kinetic steps. In this model, if a plot of q_t_ vs t^0.5^ is linear and passes through the origin, then intraparticle diffusion (IPD) occurs in the adsorption process and is considered to be the rate-limiting process [57,58,59]. In Figure 3a, the nonlinear curves and nonzero intercepts show that intraparticle diffusion is not the only rate-limiting step in the adsorption of THMs onto PVC. Instead, the adsorption involves a series of steps, such as film diffusion to the external surface, intra-particle diffusion through the pores, and rapid chemisorption at the active sites [25,58,60]. The initial sharp slope of q_t_ vs t^0.5^ from piece-wise linear regression represents the initial phase of film and intraparticle diffusion. During this phase, a higher k_p_ and lower C imply a faster diffusion rate and a reduced boundary layer effect when the adsorption sites on the PVC particles were abundant, and the initial THM concentrations in the aqueous phase were relatively high. The gentle slope of q_t_ vs. t^0.5^ afterward suggests a final equilibrium stage is approaching.

However, as a simplified model, IPD may not completely describe this adsorption behavior. Since the lines in the IPD model do not pass through the origin, this model suggests that both film diffusion and intraparticle diffusion may play roles in governing the rate-limiting sorption step. Considering that the reactors were continuously agitated in this study, the resulting rapid film diffusion of the THMs from water to the external surfaces of the microplastics was unlikely to be the rate-controlling step [58]. Hence, intraparticle diffusion may still be considered as the dominant rate-limiting step.

The Boyd kinetic model was also applied to the kinetics data to identify a possible rate-limiting step in the adsorption process of the THMs onto the PVC microplastics. (Figure 3b). When intraparticle diffusion is the rate-limiting step, a plot of B_t_ versus t should be a straight line passing through the origin; otherwise, the adsorption process is governed by film diffusion or external mass transport [25,61,62]. As shown in Figure 3b and Table S7, intraparticle diffusion is suggested as being the dominant rate-limiting step in the first 21 days, with a high linearity and an intercept close to zero. After 21 days, the more randomly distributed Boyd numbers resulted from different equations for calculating B_t_ when F fluctuated around 0.85, indicating that the adsorption was approaching equilibrium. Therefore, different kinetic models were in good agreement that the adsorption of THMs onto PVC involved film and intra-particle diffusion, with the latter being the dominant rate-limiting step.

Also considered in this study was the diffusion–chemisorption (DC) kinetic model, developed by Clint Sutherland and Chintanapalli Venkobachar (2010) to empirically simulate the sorption of ionic substances onto heterogeneous media [63]. It also demonstrated satisfactory agreement with the experimental data for THM adsorption onto PVC (Figure S6). However, since the diffusion–chemisorption model was developed for the adsorption of ionic heavy metals onto heterogeneous materials within a short time frame, it may not be entirely applicable for the slower adsorption process of organic disinfection by-products (DBPs) onto microplastics, which reached equilibrium after 21 days and are more fully included in the Supplementary Material for possible future reference with more ionic DBPs. For THMs, although the model indicated chemisorption as the dominant mechanism after three days, further analytical methods are required to validate the specific adsorption mechanisms involved.

### 3.3. Adsorption Isotherms for Virgin PVC MPs

Isotherm tests were conducted for 21 days based on the kinetic test results. Although true equilibrium might take longer to achieve, 21 days is sufficient for investigating the adsorption behavior of THMs onto microplastics in drinking water, particularly since the typical water age from a treatment facility to users is less than seven days [64]. PVC-based microplastics adsorbed 5–85% of the THMs from water, with an initial THM concentration of 100 µg L^−1^ in the adsorption isotherm tests using a PVC dose range of 0.2–10 g L^−1^. The results indicated that PVC microplastics can adsorb THMs at realistic concentration levels (<100 µg L^−1^) in actual drinking water. These findings are in reasonable agreement with a previous study by Cook and Hartz (1983), which reported that about 30% of TCMs were adsorbed onto 12 mm PVC tubing pieces with an initial TCM concentration of 100 µg L^−1^ [65].

**Figure 4:**
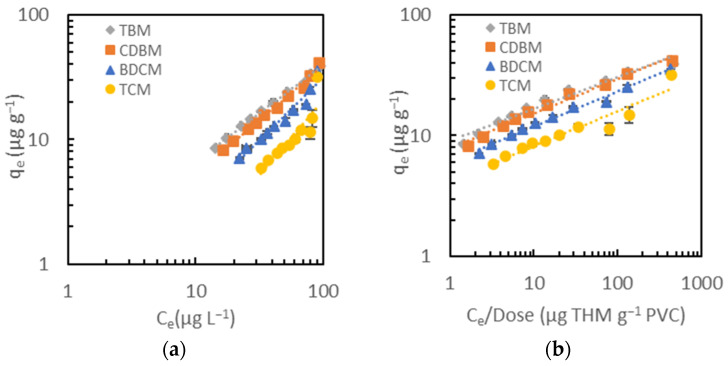
Experimental data and fitted curves for adsorption of THMs onto PVC with (**a**) Freundlich and (**b**) modified Freundlich isotherm models. Error bars represent experimental errors for duplicate analyses of duplicate samples.

The linear, Langmuir, Freundlich, and modified Freundlich isotherm models were applied to fit the data to understand the adsorption behavior of the THMs onto the PVC microplastics. The linear model, also known as Henry’s isotherm model, describes the simple solute partitioning between solid and aqueous phases [17]. The Freundlich model is an empirical equation for mono or multilayer adsorption onto a solid phase [25,66], while the Langmuir model is generally used to describe monolayer chemisorption processes [25]. In addition, the Langmuir model assumes that the adsorbent surface is homogeneous and has comparable and energetically equivalent adsorption sites [47]. These well-established isotherm models are commonly used for adsorption experiments with a relatively constant solid concentration and varying initial adsorbate concentrations [16,25,66,67,68]. However, the PVC dose was the changing variable in this adsorption experiment because, in an actual water sample, the variation in DBP concentrations that is typically experienced in DWTPs (i.e., +/− a few percent) is relatively constant when compared with the range of ratios of dissolved contaminants to adsorbent concentrations that are typically used in conventional adsorption tests (a few orders of magnitude). Thus, the modified Freundlich model was also employed, accounting for a variable adsorbent dose [48,49]. Figure 4 shows the extent to which the experimental data fit the original and modified Freundlich models, which were the models that best fit the experimental data, and Table 2 lists the values of the regression parameters of all four of the isotherm models examined in this study. For completeness, Figure S7 shows all the isotherm models mentioned for each THM compound.

The empirical modified Freundlich model provided the best fit (R^2^ in the range of 0.904–0.990) for the adsorption of all four THM compounds onto the PVC microplastics among all the isotherm models applied. As shown in Table 2, the linear and Langmuir models only fitted well for CBDM and TBM with R^2^ > 0.97, and the Freundlich model described three THM compounds with R^2^ > 0.91 (except for TCM, which, unfortunately, is the most abundant THM species in most chlorinated waters). The nonlinearity of TCM and BDCM in the linear and Langmuir models may be explained by the lower hydrophobicity of these two compounds (see physical parameters in Table S6). The error was also possibly due to the difficulty in accurately measuring their concentration changes at lower doses of PVC (< 2 g L^−1^) because of the high volatility of TCM and BDCM.

Similar to the adsorption kinetics test, the isotherm data also revealed that hydrophobic interactions are the primary mechanism for the adsorption of THMs onto PVC. In the modified Freundlich model, the Freundlich adsorption capability constants (K′_F_) increased with an increased hydrophobicity. The highest K′_F_ of TBM implied that TBM was the best adsorbed onto PVC, followed by CDBM, BDCM, and TCM [25].

Finally, it should be acknowledged that the applicability of adsorption models is constrained by the complexity of their adsorption behaviors and the accuracy of experimental adsorption data. For example, the heterogeneous nature of microplastics’ surfaces could result in deviations from the Langmuir model. Additionally, although the original and modified Freundlich models are empirical, traditionally linked to multilayer adsorption, and not typically associated with chemisorption, they may still be applicable in certain scenarios of chemisorption, particularly where surface heterogeneity plays an important role. Isotherm models by themselves may not fully elucidate the adsorption mechanisms. This study considers the possibility of both physical adsorption onto microplastic surfaces and chemical interactions with analogous functional groups in PVC and THMs.

### 3.4. Impact of Weathering on Adsorption

The weathering of microplastics can lead to a higher adsorption capacity attributable to an increased surface roughness and area compared to virgin particles. However, the increased hydrophilicity resulting from additional oxygen functional groups on weathered microplastics’ surfaces may decrease their adsorption of hydrophobic compounds. Hence, there is a need to examine the influence of PVC weathering on the adsorption of THMs.

**Figure 5:**
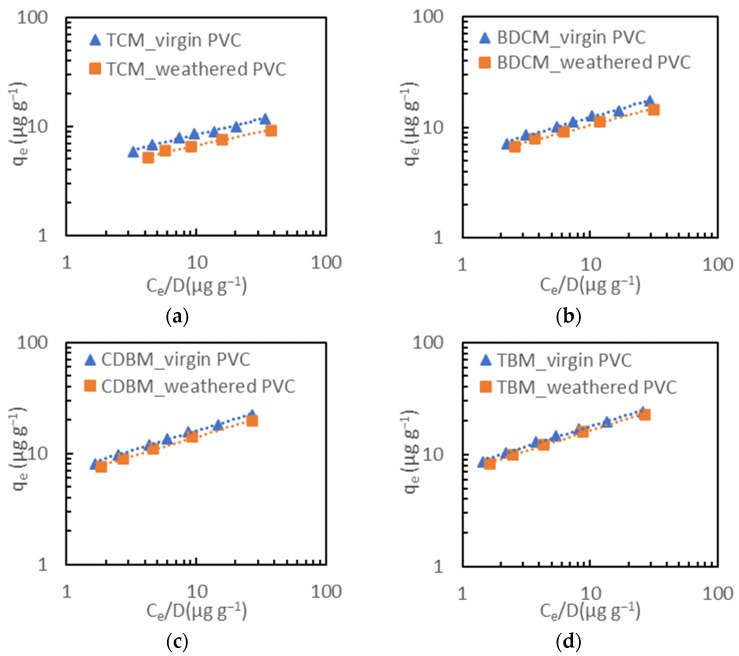
Experimental data and modified Freundlich model fitted curves for THM adsorption onto virgin and weathered PVC: (**a**) TCM, (**b**) BDCM, (**c**) CDBM, and (**d**) TBM.

The adsorption isotherms of THMs onto virgin and weathered PVC MPs were compared by a two-tailed paired t-test at equal doses. The results (Table S8) indicated a statistically significant difference (*p* < 0.05) in the THM adsorption between the two types of PVC MPs for all four compounds. The weathered PVC showed a lower adsorption capacity of THMs by approximately 10% compared to the virgin PVC. The adsorption was then evaluated by the modified Freundlich isotherm model (Table 3 and Figure 5). The modified Freundlich constants K’_F_ decreased consistently for all four THM compounds’ adsorption on the weathered PVC MPs, signifying a reduced adsorption capacity of THMs onto weathered particles. This finding aligns with the mechanism described earlier, wherein hydrophobic interactions play a primary role in THM adsorption onto PVC. The decrease in the hydrophobicity of the weathered PVC consequently led to a decline in their THM adsorption capability.

However, among the THM species, TCM, being the most hydrophilic, exhibited the most decreased adsorption on the weathered PVC. This implied that hydrophobic interactions might not be the sole adsorption mechanism. The interaction between similar structural elements, i.e., C-H and C-Cl bonds, that exist in both THM and PVC molecules, may also play an important role. With the C-H and C-Cl bonds replaced by C=O and C-O bonds and the Cl/C ratio decreased on the surfaces of the weathered particles, the adsorption of TCM was most affected, since there were less opportunities for these bond interactions. The interaction between chemical bonds may require further investigation.

The weathering process affected the surface morphology, mechanical properties, and physiochemical properties of the microplastics in this study. The weathered PVC exhibited a decreased adsorption of THMs by 10%, mainly due to the lower hydrophobicity of its surface and the associated weakened bond interactions. While our study simulated sunlight exposure and mechanical abrasion in source water in the weathering process, common water treatment processes such as UV and chlorine disinfection may have additional effects on microplastics in treated water. Prior research employing alternative aging methods which involve UV-light and strong oxidants such as K_2_S_2_O_8_ have reported enhanced changes in surface morphology, particle size, oxygen content, molecular structure, surface potential, and crystallinity [30,53,69]. In contrast to our results, Zhu et al. (2023) observed a higher adsorption of TBM onto weathered PET MPs after ozonation and chlorination treatments [70]. This discrepancy implies that, when stronger oxidants are used, the effect of an increased surface area due to the further fragmentation of MPs may outweigh the effect of a decreased hydrophobicity from the weathering process, thus enhancing the adsorption capacity overall. Therefore, further investigation is needed to understand the adsorption of disinfection byproducts (DBPs) onto real weathered microplastics.

This work showed that disinfection byproducts such as THMs may adsorb onto virgin and weathered microplastics through hydrophobic interactions and molecular bond interactions. Understanding the sorption of DBPs onto microplastics is essential for further evaluating the risks associated with microplastics in treated water for both the human body and aquatic life. Although the risk of exposure of THMs adsorbed onto microplastics is relatively low, as shown in this ‘proof of concept’ study, subsequent studies will be needed to investigate the adsorption phenomenon with multiple MPs and different DBP types in various water matrixes such as chlorinated or chloraminated waters. Further investigation is necessary to assess the influence of external factors, including temperature, pH, salinity, and water phase composition. In addition, similar interactions with more toxic disinfection byproducts (DBPs) and other types of microplastics should be explored.

## 4. Conclusions

This study investigated the adsorption behavior of THMs, byproducts of chlorinated water, onto virgin and weathered PVC microplastic particles using a range of kinetic and isotherm models. Both pseudo-first-order and pseudo-second-order models fit the kinetic data well and described the adsorption rate effectively. The intraparticle diffusion and Boyd kinetic model revealed that the adsorption of THMs onto PVC occurred in multi-stages: film diffusion and intraparticle diffusion, with the latter being the dominant rate-limiting step. In terms of isotherm models, while the linear, Langmuir, original, and modified Freundlich isotherm models could all be applied, the modified Freundlich isotherm model best described the adsorption of THMs onto PVC microplastics. In general, the kinetic and isotherm models that were applied in this study provided partial insights into adsorption mechanisms, suggesting the possibility of both physisorption and chemisorption. To confirm the adsorption mechanisms, additional analytical techniques and thermodynamic analysis are required. The surface morphology and functional groups of the microplastics were altered by weathering processes, which resulted in an overall 10% decrease in the adsorption of THMs. This suggested that the adsorption process was possibly influenced by both hydrophobic interactions and C-Cl bond interactions. In summary, this study showed that disinfection byproducts such as THMs can adsorb onto PVC microplastics, a common material used in drinking water distribution systems and sewer pipes, suggesting the necessity for further investigation of similar interactions with other DBPs and other microplastic types.

## Figures and Tables

**Table 1:** Kinetic parameters for the adsorption of THMs onto PVC microplastics.

THM Compound	Pseudo First Order (Non-Linear)			Pseudo Second Order (Non-Linear)		
	q_e_ (µg g^−1^)	K_1_ (day^−1^)	R^2^	q_e_ (µg g^−1^)	K_2_ (g(µg day)^−1^)	R^2^
TCM	9.0 ± 0.5	0.076 ± 0.004	0.845	9.0 ± 0.4	0.082 ± 0.008	0.910
BDCM	13.6 ± 0.6	0.029 ± 0.002	0.947	13.6 ± 0.6	0.031 ± 0.003	0.971
CDBM	16.5 ± 0.5	0.030 ± 0.001	0.945	16.4 ± 0.5	0.032 ± 0.001	0.976
TBM	19.3 ± 0.3	0.021 ± 0.001	0.963	19.2 ± 0.3	0.022 ± 0.000	0.984

**Table 2:** Parameters calculated with linear, Langmuir, Freundlich, and modified Freundlich isotherm models for the adsorption of THMs onto PVC microplastics in the dose range of 0.2–10 g L^−1^.

Isotherm Model	THM Compound	Parameters		
		K (L g^−1^)	Intercept	R^2^
Linear isotherm model (Henry)	TCM	0.30	−5.85	0.618
	BDCM	0.35	−1.92	0.899
	CDBM	0.40	1.40	0.974
	TBM	0.36	4.39	0.993
		K_L_	q_max_ (µg g^−1^)	R^2^
Langmuir (nonlinear form)	TCM	0.00012	1766	0.553
	BDCM	0.00007	4798	0.889
	CDBM	0.00060	730	0.970
	TBM	0.00558	108	0.990
		K_F_	1/n	R^2^
Freundlich (nonlinear form)	TCM	0.01	1.86	0.669
	BDCM	0.13	1.21	0.910
	CDBM	0.52	0.95	0.971
	TBM	1.11	0.78	0.994
		K′	1/n′	R^2^
Modified Freundlich (nonlinear form)	TCM	1.91	0.36	0.904
	BDCM	4.17	0.29	0.990
	CDBM	6.06	0.26	0.987
	TBM	7.51	0.23	0.963

**Table 3:** Modified Freundlich isotherm model constants for the adsorption of THMs onto virgin and weathered PVC microplastics.

THM Compound	PVC	K′	1/n′	R^2^
TCM	Virgin	4.326	0.287	0.989
	Weathered	3.710	0.253	0.991
BDCM	Virgin	5.693	0.332	0.992
	Weathered	5.116	0.308	0.995
CDBM	Virgin	7.059	0.356	0.994
	Weathered	6.244	0.353	0.996
TBM	Virgin	7.823	0.355	0.996
	Weathered	7.082	0.363	0.997

## Data Availability

Dataset available on request from the authors.

## Supplementary Materials

The following supporting information can be downloaded at: https://www.mdpi.com/article/10.3390/toxics12070450/s1, Table S1: Comparison of THMs adsorption onto PE in the adsorption trial* and the estimated adsorption based on the results reported by Cao et al. (2020). Figure S1: THM concentrations after adsorption onto 90 µm PP, 250 µm PA, PAN, and PET microplastics for 4 days with 4 g L^−1^ microplastic dose and 100 µg L^−1^ initial aqueous phase THM concentrations. Error bars represent experimental variability for duplicate analyses of duplicate samples. Figure S2: Microscopic images of polymer surfaces, a). PVC, b).PE, c). PP, d). PA, e). PAN, f). PET. Figure S3. Concentrations of THMs in bottles containing 0.1, 2.5, and 25 mg L-1 NaN_3_ after 4 and 18 days with error bars representing analytical error in duplicate sample analysis by GC-ECD. The target initial concentration was 50 µg L^−1^. Table S2: Comparison of adsorption capacity of each THM compound onto PVC with or without NaN_3_ for 14 days using two-factor ANOVA with replication (α < 0.05). Table S3: Summary of the kinetic models used in this study. Table S4: Summary of the isotherm models used in this study. Table S5: Elemental analysis of virgin and weathered PVC by XPS. Figure S4: (a) FTIR-ATR absorbance spectra of virgin and weathered PVC, (b) FTIR-ATR spectra absorbance of C=O for virgin and weathered PVC, and (c) FTIR-ATR spectra absorbance of C-O for virgin and weathered PVC. Table S6: Solubility, Henry’s law constants and typical concentrations of THMs in drinking water. Table S7: Intraparticle diffusion model, Boyd kinetic model, and diffusion-chemisorption model kinetic parameters for sorption of THMs onto PVC microplastics at 4 g L^−1^. Figure S5: THM concentration change after adsorption onto PVC microplastics for 35 days with 4 g L^−1^ microplastic dose and 100 µg L^−1^ initial THM concentrations. Error bars represent experimental variability for duplicate analyses of duplicate samples. Figure S6: Experimental data and fitted curves for the adsorption of THMs onto PVC with diffusion-chemisorption kinetic model. Error bars represent experimental errors for duplicate analyses of duplicate samples, and the oval identifies the inflection points on Day 3 in the diffusion-chemisorption model. Figure S7: Experimental data and fitted curves with linear, Langmuir, Freundlich, and modified Freundlich isotherm models for adsorption of TCM (a), BDCM (b), CDBM (c), and TBM (d) onto PVC. Error bars represent experimental errors for duplicate analyses of duplicate samples. Table S8: Comparison of THM adsorption isotherm onto virgin and weathered PVC using a two-tailed paired *t*-test at equal doses [71,72].
