# Supplementary material for: Adsorption Behavior and Mechanisms of Trihalomethanes onto Virgin and Weathered Polyvinyl Chloride Microplastics
Source: Toxics. 2024 Jun 22;12(7):450. doi: 10.3390/toxics12070450 (PMC11281136; doi:10.3390/toxics12070450)
Supplement: Supplementary file 1 [file toxics-12-00450-s001.zip › toxics-3062703-supplementary.pdf]

Supplementary Materials for

Adsorption behavior and mechanisms of trihalomethanes onto virgin and weathered polyvinyl chloride microplastics

Yi Li*, Paragi Neema, Susan Andrews

Department of Civil and Mineral Engineering, University of Toronto, 35 St. George Street, Toronto, Ontario, M5S 1A4, Canada

* Corresponding Author

Yi Li,

Department of Civil and Mineral Engineering, University of Toronto, Toronto, Ontario, Canada M5S 1A4;

Email: livia.li@mail.utoronto.ca

Table S1. Comparison of THMs adsorption onto PE in the adsorption trial* and the estimated adsorption based on the results reported by Cao et al. (2020)

	TCM qe (μg g^{-1})	BDCM qe (μg g^{-1})	CDBM qe (μg g^{-1})	TBM qe (μg g^{-1})	Estimated q_e of THMs from Cao et al. (2020) q_e (μg g^{-1})
Dose 1(120g L^{-1})	0.081	0.090	0.094	0.098	-
Dose 2 (180g L^{-1})	0.074	0.080	0.084	0.087	-
Dose 3 (240g L^{-1})	0.101	0.103	0.100	0.101	0.083

* Adsorption for 3 days with C_0= 50 μg L^{-1}

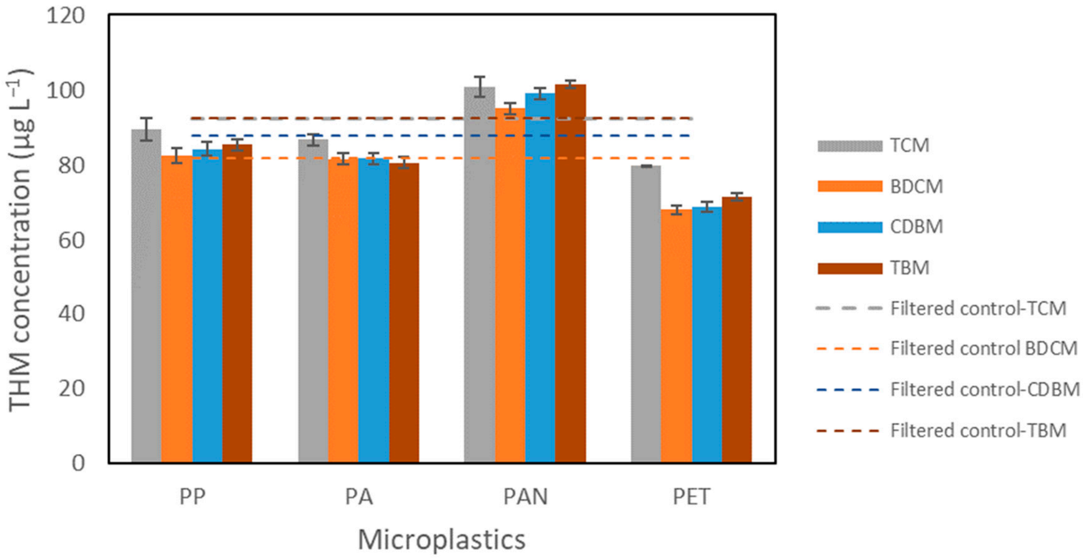

Figure S1. THM concentrations after adsorption onto 90 μm PP, 250 μm PA, PAN, and PET microplastics for 4 days with 4 g L^{-1} microplastic dose and 100 μg L^{-1} initial aqueous phase THM concentrations. Error bars represent experimental variability for duplicate analyses of duplicate samples.

Microscopy images at 90 times magnification show other polymer particles have more smooth surfaces than PVC particles (Figure S2), which may partially explain the higher adsorption of THMs onto PVC microplastics.

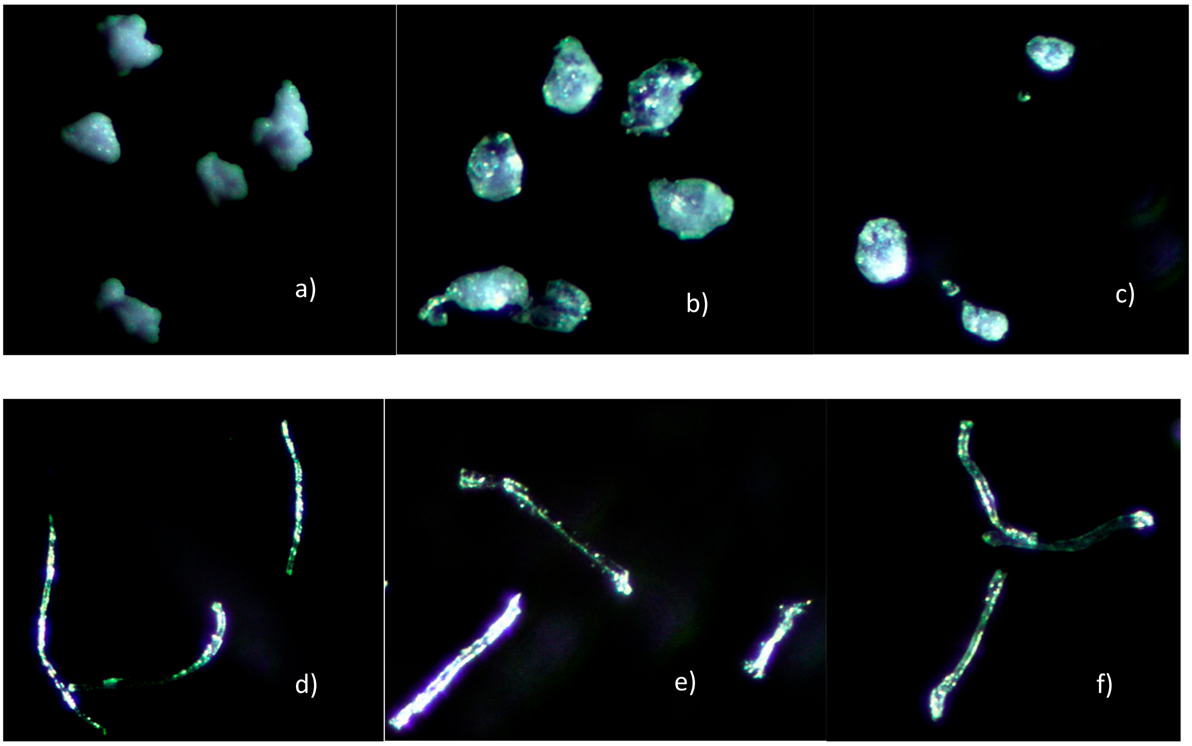

Figure S2. Microscopic images of polymer surfaces, a). PVC, b).PE, c). PP, d). PA, e). PAN, f). PET.

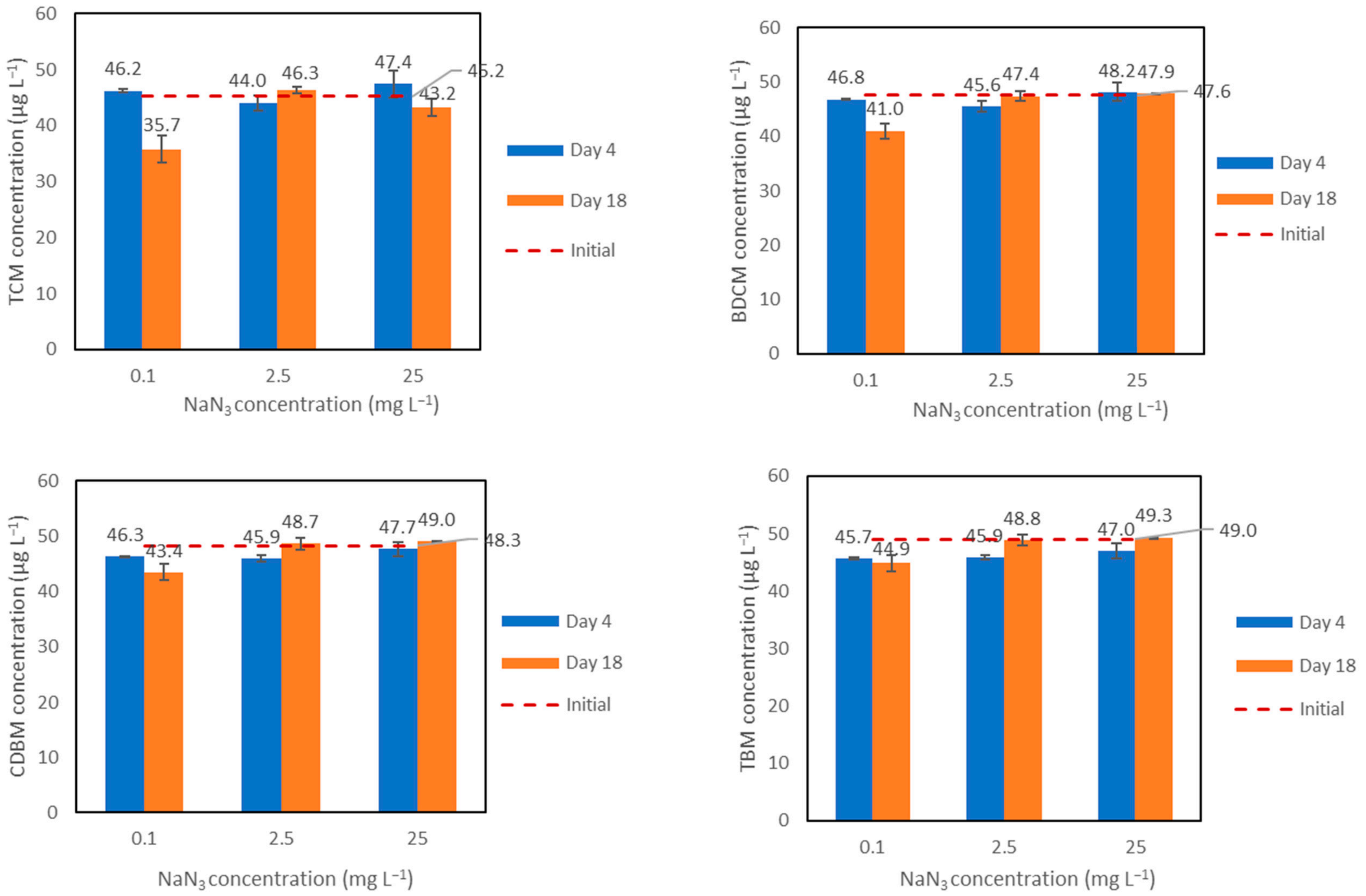

Figure S3. Concentrations of THMs in bottles containing 0.1, 2.5, and 25 mg L^{-1} NaN_3 after 4 and 18 days with error bars representing analytical error in duplicate sample analysis by GC-ECD. The target initial concentration was 50 µg L^{-1}.

The adsorption of each THM species onto PVC with and without NaN_3 was compared by 2-factor ANOVA with replication. The results suggested no significant difference between the samples with or without NaN_3 in the water matrixes. However, a significant ($\alpha < 0.05$) difference between the adsorption capacity of each THM compound was observed. See p-values and F-values in Table S2.

Table S2. Comparison of adsorption capacity of each THM compound onto PVC with or without NaN_3 for 14 days using 2-factor ANOVA with replication ($\alpha < 0.05$)

ANOVA	*F*	*P-value*	*F crit*	*Note*
Effect of NaN_3	1.56	0.21	3.90	p> 0.05, no significant difference
THM compound	9.17	0.00001	2.66	P<0.05, significantly different
Interaction	0.01	1.00	2.66	

Table S3. Summary of the kinetic models used in this study

Kinetic model	Equation	Linear form	Reference
Pseudo-first-order	$\frac{dq_t}{dt} = k_1(q_e - q_t)$	$\ln(q_e - q_t) = \ln q_e - k_1 t$	Ho and McKay, 1998
Pseudo-second-order	$\frac{dq_t}{dt} = k_2(q_e - q_t)^2$	$\frac{t}{q_t} = \frac{1}{k_2 q_e^2} + \frac{1}{q_e} t$	Ho and McKay, 1998
Intraparticle diffusion	$q_t = k_p t^{0.5} + C$	-	Morris and Weber, 1964
Boyd kinetic model	If F ≤ 0.85, $B_t = (\sqrt{\pi} - \sqrt{\pi - \left(\frac{\pi^2 F}{3}\right)})^2$, If F > 0.85, $B_t = -0.4977 - ln(1 - F)$	-	Boyd et al., 1947
Diffusion-chemisorption	$\frac{t^{0.5}}{q_t} = \frac{1}{K_{DC}} + \frac{1}{q_e} t^{0.5}$	-	Sutherland and Venkobachar, 2010

q_e = the amounts of each THM compound adsorbed (μg g^{-1}) at equilibrium

q_t = the amounts of each THM compound adsorbed (μg g^{-1}) at time t

k_1 = the pseudo-first-order adsorption rate constant

k_2 = the pseudo-second-order adsorption rate constant

k_p = the internal diffusion rate constant

C = the constant related to the boundary-layer thickness

F = $\frac{q_t}{q_e}$, the fraction of THM adsorbed at time t

B_t = Boyd number as a mathematical function of F

K_{DC} = the diffusion-chemisorption rate constant

Table S4. Summary of the isotherm models used in this study

Isotherm model	Equation	Linear form	Reference
Linear (Henry)	$q_e = k_D C_e$	$q_e = k_D \times C_e$	Limousin et al., 2007
Freundlich	$q_e = k_F C_e^{1/n}$	$\log q_e = \log k_F + \frac{1}{n}\log C_e$	Limousin et al., 2007
Langmuir	$q_e = \frac{q_{max} k_L C_e}{1 + k_L C_e}$	$\frac{q_e}{C_e} = q_{max} k_L - k_L q_e$	Limousin et al., 2007
Modified Freundlich	$q_e = k'_F (\frac{C_e}{D})^{1/n'}$	$\log q_e = \log k'_F + \frac{1}{n'}\log(\frac{C_e}{D})$	Summers and Roberts, 1988

k_D = partition coefficient between PVC and THMs at equilibrium

C_e = THM concentrations in the liquid phase at equilibrium

q_{max} = maximum adsorption capacity

k_L = Langmuir constant

k_F and n = Freundlich constants

k'_F and n' = modified Freundlich constants

Table S5. Elemental analysis of virgin and weathered PVC by XPS

Element	Virgin PVC	Weathered PVC
**C**	68.6%	61.4%
**Cl**	16.5%	14.3%
**O**	14.7%	19.4%
Si	0.2%	2.2%
Al	0	1.5%
Fe	0	0.2%
N	0	0.8%
Ca	0	0.2%
O/C ratio	0.21	0.32
Cl/C ratio	0.24	0.23

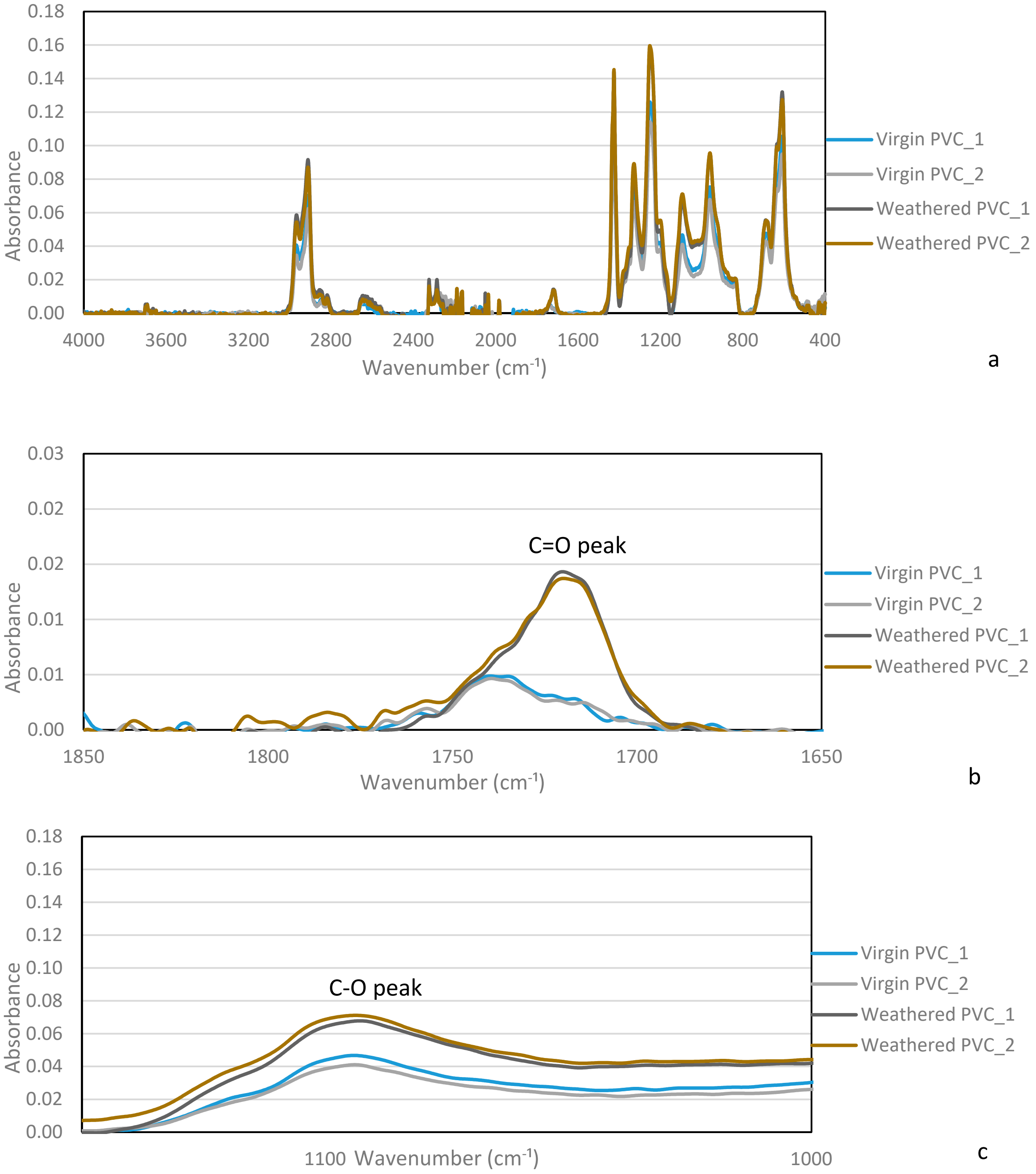

Figure S4. (a) FTIR-ATR absorbance spectra of virgin and weathered PVC, (b) FTIR-ATR spectra absorbance of C=O for virgin and weathered PVC, and (c) FTIR-ATR spectra absorbance of C-O for virgin and weathered PVC

Table S6: Solubility, Henry's law constants and typical concentrations of THMs in drinking water

THM name	Molecular formula	Molecular weight (g mol^{-1})	Solubility in water at 20 °C (g L^{-1})	Henry's Law constant (dimensionless)[c]	Typical concentration levels in drinking water (µg L^{-1})	Maximum acceptable concentration (MAC)	Reference
chloroform (TCM)	$CHCl_3$	119.4	8[a]	0.128	< 50	100 µg L^{-1} for total THMs, 16 µg L^{-1} for BDCM	Health Canada (HC), 2009
bromodichloromethane (BDCM)	$CHBrCl_2$	163.8	4.5[a]	0.0728	< 10		
chlorodibromomethane (CDBM)	$CHClBr_2$	208.3	2.7[b]	0.0371	< 10		
bromoform (TBM)	$CHBr_3$	252.7	1[a]	0.0167	< 0.5		

a. Solubility obtained from International Chemical Safety Cards (ICSCs): https://www.ilo.org/dyn/icsc/showcard.listCards3
b. Solubility obtained from PubChem Hazardous Substances Data Bank (HSDB): https://pubchem.ncbi.nlm.nih.gov/compound/31296#section=Solubility
c. Henry's Law constants were calculated by Washington (1996) method: https://www3.epa.gov/ceampubl/learn2model/part-two/onsite/esthenry.html

Table S7. Intraparticle diffusion model, Boyd kinetic model, and diffusion-chemisorption model kinetic parameters for sorption of THMs onto PVC microplastics at 4 g L^{-1}

Kinetic model	THM Compound	Parameters		
		K_p(µg (g $day^{0.5})^{-1}$)	C (µg g^{-1})	R^2
Intraparticle diffusion Day 0-11	TCM	1.89	2.14	0.844
	BDCM	3.08	1.43	0.948
	CDBM	3.64	2.55	0.934
	TBM	4.38	2.04	0.958
Intraparticle diffusion Day 11-35	TCM	0.63	5.48	0.498
	BDCM	0.70	8.89	0.736
	CDBM	0.95	10.67	0.900
	TBM	1.09	12.15	0.932
		Slope	Intercept	R^2
Boyd Day 0-21	TCM	0.07	0.23	0.895
	BDCM	0.10	0.06	0.986
	CDBM	0.11	0.09	0.995
	TBM	0.11	0.04	0.997
		K_{DC}(µg (g $day^{0.5})^{-1}$)	q_e (µg g^{-1})	R^2
Diffusion-chemisorption Day 3-35	TCM	8.31	10.67	0.928
	BDCM	7.43	14.13	0.939
	CDBM	10.23	22.10	0.993
	TBM	10.94	26.12	0.994

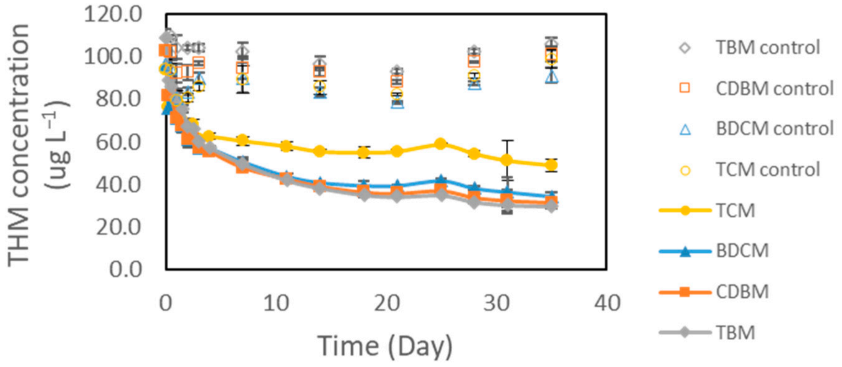

Figure S5. THM concentration change after adsorption onto PVC microplastics for 35 days with 4 g L^{-1} microplastic dose and 100 μg L^{-1} initial THM concentrations. Error bars represent experimental variability for duplicate analyses of duplicate samples.

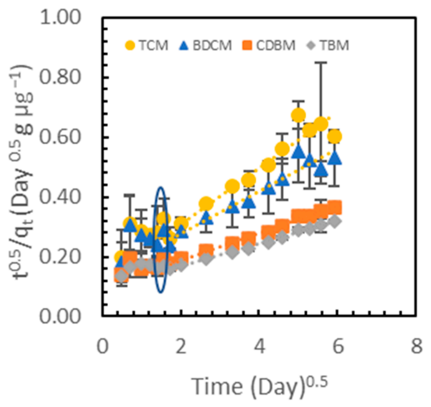

Figure S6. Experimental data and fitted curves for the adsorption of THMs onto PVC with diffusion-chemisorption kinetic model. Error bars represent experimental errors for duplicate analyses of duplicate samples, and the oval identifies the inflection points on Day 3 in the diffusion-chemisorption model.

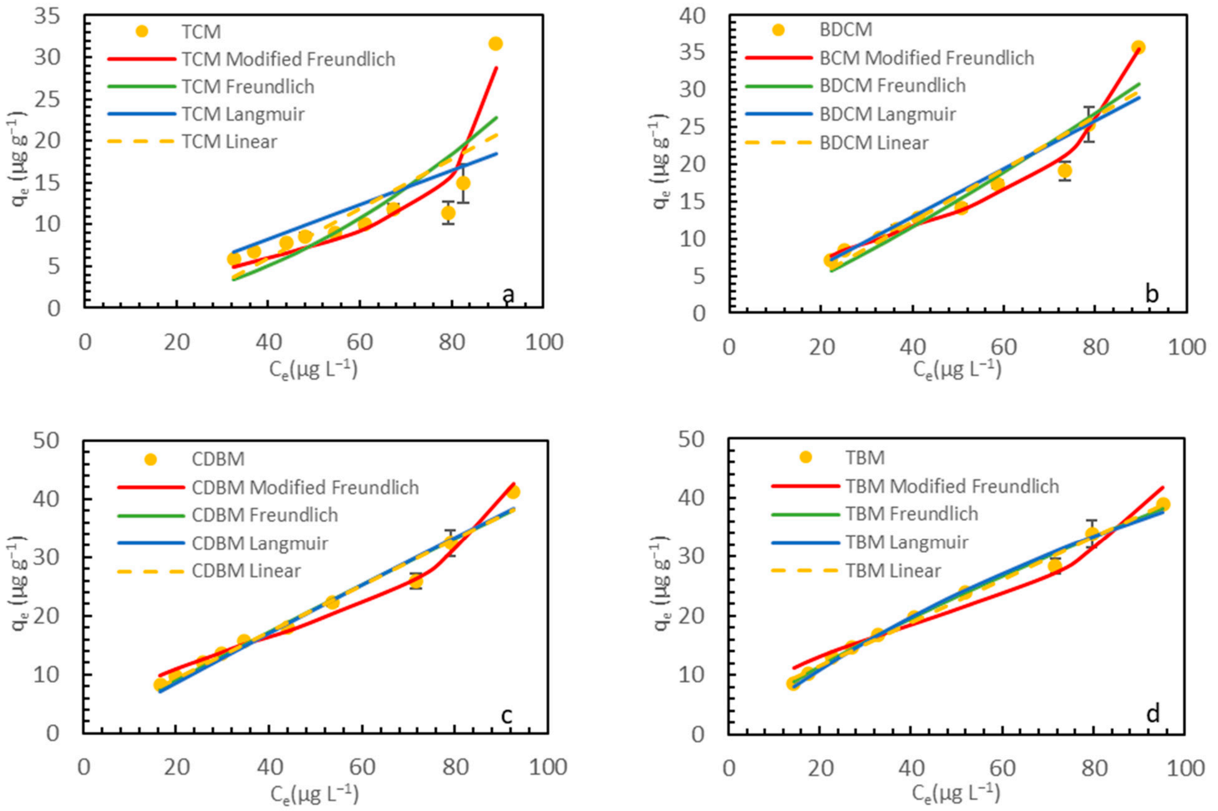

Figure S7**.** Experimental data and fitted curves with linear, Langmuir, Freundlich, and modified Freundlich isotherm models for adsorption of TCM (a), BDCM (b), CDBM (c), and TBM (d) onto PVC. Error bars represent experimental errors for duplicate analyses of duplicate samples.

Table S8. Comparison of THM adsorption isotherm onto virgin and weathered PVC using a two-tailed paired t-test at equal doses

THM compound	p value
TCM	0.005
BDCM	0.035
CDBM	0.020
TBM	0.015

References

1. Cao, G.; Huang, K.; Whelton, A.J.; Shah, A.D. Formation and sorption of trihalomethanes from cross-linked polyethylene pipes following chlorinated water exposure. *Environ. Sci. Water Res. Technol.* **2020**, *6*, 2479–2491. https://doi.org/10.1039/d0ew00262c.
2. Ho, Y.S.; McKay, G. A Comparison of chemisorption kinetic models applied to pollutant removal on various sorbents. *Process Saf. Environ. Prot.* **1998**, *76*, 332–340. https://doi.org/10.1205/095758298529696.
3. Clint Sutherland and Chintanapalli Venkobachar, A diffusion-chemisorption kinetic model for simulating biosorption using forest macro-fungus, fomes fasciatus. *Int. Res. J. Plant Sci.* 2010. Available online: http://interesjournals.org/irjps/october-2010-vol-1-issue-4/a-diffusion-chemisorption-kinetic-model-for-simulating-biosorption-using-forest-macrofungus-fomes-fasciatus (accessed on 9 August 2021).
4. Morris, J.C.; Weber, W.J. Removal of biologically-resistant pollutants from water waters by adsorption. In *Advances in Water Pollution Research*; Elsevier: Amsterdam, The Netherlands, 1964; pp. 231–266. https://doi.org/10.1016/b978-1-4832-8391-3.50032-4.
5. Boyd, G.E.; Adamson, A.W.; Myers, L.S. The Exchange Adsorption of Ions from Aqueous Solutions by Organic Zeolites. II.; Kinetics. *J. Am. Chem. Soc.* **1947**, *69*, 2836–2848. https://doi.org/10.1021/ja01203a066.
6. G. Limousin, J.P. Gaudet, L. Charlet, S. Szenknect, V. Barthès, M. Krimissa, Sorption isotherms: A review on physical bases, modeling and measurement, *Appl. Geochemistry* **2007**, *22,* 249–275. https://doi.org/10.1016/j.apgeochem.2006.09.010.
7. Summers, R.S.; Roberts, P.V. Activated carbon adsorption of humic substances. I. Heterodisperse mixtures and desorption. *J. Colloid Interface Sci.* **1988**, *122*, 367–381. https://doi.org/10.1016/0021-9797(88)90372-4.
8. Health Canada. *Guidelines for Canadian Drinking Water Quality: Guideline Technical Document — Trihalomethanes*; Health Canada: Ottawa, Canada. 2009. Available online: https://www.canada.ca/content/dam/canada/health-canada/migration/healthy-canadians/publications/healthy-living-vie-saine/water-trihalomethanes-eau/alt/water-trihalomethanes-eau-eng.pdf (accessed on 4 July 2021).
